# Modulation of PPARγ Provides New Insights in a Stress Induced Premature Senescence Model

**DOI:** 10.1371/journal.pone.0104045

**Published:** 2014-08-07

**Authors:** Stefania Briganti, Enrica Flori, Barbara Bellei, Mauro Picardo

**Affiliations:** Laboratory of Cutaneous Physiopathology, San Gallicano Dermatologic Institute, Istituto di Ricovero e Cura a Carattere Scientifico, Rome, Italy; University of Tennessee, United States of America

## Abstract

Peroxisome proliferator-activated receptor gamma (PPARγ) may be involved in a key mechanism of the skin aging process, influencing several aspects related to the age-related degeneration of skin cells, including antioxidant unbalance. Therefore, we investigated whether the up-modulation of this nuclear receptor exerts a protective effect in a stress-induced premature senescence (SIPS) model based on a single exposure of human dermal fibroblasts to 8-methoxypsoralen plus + ultraviolet-A-irradiation (PUVA). Among possible PPARγ modulators, we selected 2,4,6-octatrienoic acid (Octa), a member of the parrodiene family, previously reported to promote melanogenesis and antioxidant defense in normal human melanocytes through a mechanism involving PPARγ activation. Exposure to PUVA induced an early and significant decrease in PPARγ expression and activity. PPARγ up-modulation counteracted the antioxidant imbalance induced by PUVA and reduced the expression of stress response genes with a synergistic increase of different components of the cell antioxidant network, such as catalase and reduced glutathione. PUVA-treated fibroblasts grown in the presence of Octa are partially but significantly rescued from the features of the cellular senescence-like phenotype, such as cytoplasmic enlargement, the expression of senescence-associated-β-galactosidase, matrix-metalloproteinase-1, and cell cycle proteins. Moreover, the alterations in the cell membrane lipids, such as the decrease in the polyunsaturated fatty acid content of phospholipids and the increase in cholesterol levels, which are typical features of cell aging, were prevented. Our data suggest that PPARγ is one of the targets of PUVA-SIPS and that its pharmacological up-modulation may represent a novel therapeutic approach for the photooxidative skin damage.

## Introduction

Ultraviolet (UV) radiation elicits premature aging of the skin and cutaneous malignancies [1]. UVA rays generate reactive oxygen species (ROS) via photodynamic actions [2], resulting in skin degeneration and aging [3], [4] and, in particular, oxidative damage to lipids, proteins, and DNA [5]–[7]. Moreover, UVA-induced ROS regulate the gene expression of matrix metallo-proteinases (MMPs), which are the main enzymes responsible for dermal extracellular matrix degradation [8]–[10]. As a result, the incidence of skin photoaging and skin cancer dramatically increases with increased exposure to UVA rays [11]. To protect its structure against UV, skin has developed several defence systems which include pigmentation, antioxidant network and neuro-immune-endocrine functions, which are tightly networked to central regulatory system and are involved in the protection and in the maintenance of global homeostasis, through the production of cytokines, neurotransmitters, neuroendocrine hormones [12]. Thus, UV would stimulate production and secretion of α-melanocyte-stimulating hormone, proopiomelanocortin-derived β-endorphin, adrenocorticotropin, corticotrophin releasing factor, and glucocorticoids [13]. An unbalance between pro-inflammatory or anti-inflammatory responses activated by these mediators may be related to cellular degeneration in aged skin.

A way to investigate *in vitro* aging process is the study of cellular senescence, a loss of proliferative capacity attributed to telomere shortening during cell replication or after exposure to pro-oxidant stimuli and closely interconnected with aging, longevity and age-related disease [14], [15]. Due to the key role of oxidative stress in the photoaging process, the change of proliferating skin cells to photo-aged cells resembles premature senescence under conditions of artificially increased ROS levels. Consistently, stress-induced premature senescence (SIPS) models can represent useful tools with which to investigate the biological and biochemical mechanisms involved in photo-induced skin damage and photocarcinogenesis and to evaluate the potential protective effects of new molecules. SIPS can be induced in human skin dermal fibroblasts (HDFs) by a single subcytotoxic exposure to UVA-activated 8-methoxypsoralen (PUVA) [16], widely used in the treatment of different skin disorders like psoriasis, T-cell lymphoma and other inflammatory skin disorders. We previously reported that oxidative stress and cell antioxidant capacity are involved in both the induction and maintenance of PUVA-SIPS and supplementation with low-weight antioxidants abrogated the increased ROS generation and rescued fibroblasts from the PUVA-dependent changes in the cellular senescence phenotype [17]. Moreover, PUVA treatment induced a prolonged expression of interstitial collagenase/MMP-1, leading to connective tissue damage, a hallmark of premature aging [17], confirming this experimental model as a useful tool to investigate in vitro the mechanisms of skin ageing. The function of nuclear receptors has been reported to be involved in the molecular mechanisms controlling the aging process. The peroxisome proliferator-activated receptor (PPAR) family regulates the function and expression of complex gene networks, especially involved in energy homeostasis and inflammation [18]–[20], and modulate the balance between MMP activity and collagen expression to maintain skin homeostasis [21]. In particular, PPARγ has been implicated in the oxidative stress response, an imbalance between antithetic pro-oxidation and antioxidation, and in this delicate and intricate game of equilibrium, PPAR*γ* stands out as a central player specializing in the quenching and containment of damage and fostering cell survival. Moreover, PPARγ activation has been reported to restore the “youthful” structure and function of mitochondria that are structurally and functionally impaired by excessive oxidant stress [22]. However, PPARγ does not act alone, but is interconnected with various pathways, such as the nuclear factor erythroid 2-related factor 2 (NRF2), Wnt/*β*-catenin, and forkhead box protein O (FoxO) pathways [23]. PPARγ activation has been reported to be a link to melanocyte differentiation pathways, as suggested by the ability of PPARγ ligands to regulate Microphthalmia-associated transcription factor gene and Wnt/*β*-catenin levels, promoting differentiation and growth arrest of melanoma cells [24]. Given these features, PPARγ is emerging as an important regulator of skin photodamage.

Among anti-aging agents, topical all-trans-retinoic acid (AtRA) inhibits MMP expression [25] and has a significant diminishing effect on UV-induced photoaging, such as wrinkles, water loss, and reduced wound healing [26]. However, irritant reactions, such as burning, scaling, or dermatitis, limit the acceptance of AtRA by patients [27]. To minimize these side effects, various novel drug delivery systems have been developed; in addition, screening to discover new natural or synthetic retinoid-like molecules has been conducted. Psittacofulvins are a mixture of polyenals identified exclusively in the red plumage of the Ara macao [28], indicating that these compounds are produced at the feather bulb for defense against environmental insults. Parrodienes, congeners of psittacofulvins that are considered retinoid-like molecules, as they possess a polyene structure and an alcohol functional group, have been synthesized to investigate the biological effects of psittacofulvins. Studies have shown that parrodienes possess antioxidant [29] and anti-inflammatory activities and are able to inhibit the lipoperoxidation of cell membranes induced by CCl_4_ [30]. Among the parrodiene family members, 2,4,6-octatrienoic acid (Octa) promotes melanogenesis and antioxidant defense in normal human melanocytes, and its mechanism of action involves the modulation of PPARγ [31]. We added Octa to PUVA-treated HDFs to evaluate Octa's ability to counteract PUVA-SIPS and to investigate whether PPARγ is involved in photo-induced cell senescence.

## Materials and Methods

### Standards and reagents

Dulbecco's modified Eagle's medium (DMEM), penicillin and streptomycin were purchased from Gibco, Life Technologies Italia, Milan, Italy. Octa was furnished by Giuliani Pharma, Milan, Italy. Crystalline 8-methoxypsoralen (8-MOP), dimethylsulfoxide (DMSO), 3-(4,5 dimethylthiazol)-2,5-diphenyl tetrazolium bromide (MTT), butylated hydroxytoluene (BHT), 6-hydroxy-2,5,7,8-tetramethylchromane-2-carboxylic acid (Trolox), N-ethylmaleimide (NEM), thiosalicylic acid (TSA), sodium methoxide, potassium hydroxide (KOH), retinol (ReOH) and all-trans retinoic acid AtRA were from Sigma-Aldrich, Milan, Italy. 2′,7′-dichlorodihydrofluorescein diacetate (DCFH_2_-DA) was from Molecular Probes (Eugene, OR, USA). All organic solvents used were of HPLC-grade.

### Cell culture and treatments

Human Dermal Fibroblasts (HDFs) were derived from neonatal foreskin of healthy male caucasian individuals (n = 3), phototype III, ranged from 4 to 7 years old and were isolated as previously described [16]. Cells were grown in DMEM supplemented with 10% FBS, penicillin (100 U/ml) and streptomycin (100µg/ml) and used between passage 2 and 8. Institutional Research Ethic committee (Istituti Fisioterapici Ospitalieri) approval was obtained to collect sample of human material for research. The Declaration of Helsinki Principle was followed and due to the fact that the study included children participants their parents gave written informed consent. Stock solutions (10 mM) of Octa was prepared in DMSO. The maximum concentration of Octa, without affecting cell viability or proliferation, was determined by MTT assay and Trypan blue exclusion test (data not shown). Moreover we did not observe any relevant modification of protein content in Octa treated cells (data not shown).

### PUVA treatment

8-MOP (25 ng/ml) was added to the cell culture medium overnight. Cell were washed twice with phosphate-buffered saline (PBS) containing 8-MOP 25 ng/ml. HDFs were irradiated at a dose of 6 J/cm^2^ using a Bio-Sun irradiation apparatus (Vilbert Lourmat, Marnè-la-Vallée, France) with maximum emission at 365nm in the UVA spectral region (340 to 450 nm). Following irradiation, PBS was replaced by fresh medium which was changed every three days. Octa was diluted in cell culture medium at a final concentration of 2 µM and added to HDFs immediately following PUVA and twice a week thereafter.

### Cell morphology

To monitor fibroblast morphology after PUVA treatment, fibroblasts were fixed and stained with Comassie brilliant Blue as previously described [32].

### Senescence associated beta-galactosidase (SA-β-gal) staining

SA-β-gal staining was performed as previously described [33]. The proportion of cells positive for SA-β-gal activity are given as percentage of the total number of fibroblasts counted in each dish. Triplicates were performed. The stained dishes were photographed, positive fibroblasts counted and the results expressed as mean ± S.D. of SA-β-gal positive fibroblasts in% of total fibroblast number.

### MMP-1 ELISA

MMP-1 total release (proMMP-1, active MMP-1 and MMP-1/TIMP-1 complex) was measured using an Human, Biotrack ELISA immunoassay (Amersham Pharmacia Biotech, Milan, Italy), according to the manufacturer's instructions, and was normalized against protein concentration, determined by Quick Start Bradford Dye Reagent (Bio-Rad, Hercules, CA, USA). The results are the mean ± S.D. of experiments performed in each donor (n = 3) in triplicate.

### Determination of ROS generation

The generation of intracellular ROS was determined by employing the cell-permeable fluorogenic probe DCFH-DA. In brief, DCFH-DA is diffused into cells and deacetylated by cellular esterases to non fluorescent 2′, 7′-dichlorofluorescin (DCFH), which is rapidly oxidazed to highly fluorescent 2′, 7′-dichlorofluorescein (DCF). The fluorescence intensity of the supernatant was measured with a multiplate reader (DTX 880 Multimode Detector; Beckman Coulter Srl, Milan, Italy) at 485nm excitation and 535 nm emission. Cellular oxidant levels were expressed as relative DCF fluorescence per microgram of protein. The results are the mean ± S.D. of experiments performed in each donor (n = 3) in triplicate.

### JC-1 assay for mitochondrial membrane potential

Mitochondrial trans-membrane potential (ΔΨ_m_) was assessed in live HDFs using the lipophilic cationic probe 5,5′,6,6′-tetrachloro-1,1′,3,3′-tetraethylbenzimidazolcarbocyanine iodide (JC-1, Molecular Probes). For quantitative fluorescence measurements, cells were rinsed once after JC-1 staining and scanned with a Flow cytometer (FACS-Calibur, Becton Dickinson, San José, CA, USA) at 485 nm excitation, and 530 and 570 nm emission, to measure green and orange-red JC-1 fluorescence, respectively. Results of experiments performed in each donor (n = 3) in triplicate are expressed as percentage of variation (± SD) respect to control values of the orange-red/green fluorescence intensity ratio.

### Catalase (Cat) activity

Fibroblasts were lysed in PBS by repeated freezing and thawing, in the presence of protease inhibitors. Cat activity was determined by spectrophotometric monitoring the rate of disappearance of H_2_O_2_ at 240 nm [34]. A standard curve was obtained with bovine catalase (Sigma-Aldrich, Srl Milan, Italy). Units were normalized for protein content. Results of experiments performed in each donor (n = 3) in triplicate are given as% of relative units of Cat per mg protein ± S.D.

### Biological Antioxidant Potential (BAP) Assay

BAP was measured with a commercially available assay kit (Diacron srl, Grosseto Italy). The principle of the test is to measure the color change upon reduction of Fe^3+^ to Fe^2+^ by the reducing components in the sample. The optical density was measured at 505 nm by a microplate reader. The data were obtained by interpolating the absorbance on a calibration curve obtained with Trolox (30–1000 µM). Results of experiments performed in each donor (n = 3) in triplicate are expressed as medium percentage of variation (± S.D.) respect to control values of untreated cells.

### Glutathione (GSH) measurement

GSH levels were determined in cell lysates by high-performance liquid chromatography-mass spectrometry (HPLC-MS) as previously described [35]. The mean value of experiments performed in each donor (n = 3) in triplicate is given as GSH in nmol/mg of total protein ± S.D.

### Alpha-tocopherol (α-Toc) analysis

Cells were extracted in hexane:ethanol 3∶1 in the presence of γ and δ tocopherol (Sigma-Aldrich, Milan, Italy), as internal standards, and the tocopherols were analysed by gas chromatography-mass spectrometry (GC-MS) as previously described [36]. The mean value of experiments performed in each donor (n = 3) in duplicate is expressed as nanogram per milligram of proteins ± S.D.

### Assessment of cell membrane phospholipids polyunsaturated fatty acids

Cell pellets were extracted twice in chloroform/methanol (2∶1, v:v) in the presence of tricosanoic acid methyl ester (Sigma Aldrich, Milan Italy), as internal standard. Fatty acids of cell total lipid extract were analysed by GC-MS on a capillary column (FFAP, 60 m×0.32 µm×0.25 mm, Hewlett Packard, Palo Alto, CA, USA), as previously reported [36]. Results of experiments performed in each donor (n = 3) in triplicate are given as mean percentage ± S.D.

### Conjugated Dienes

Conjugated diene level was evaluated as described by Kurien and Scofield [37] with modification. Cells were extracted with 3 ml chloroform/methanol (2∶1, v/v). After centrifugation at 3,000 rpm for 15 min, 2 ml of organic phase was transferred into another tube and dried at 45°C. The dried lipids were dissolved in 2 ml of methanol and absorbance at 234 nm was determined. It corresponds to the maximum absorbance of the extracted compounds. Results of experiments performed in each donor (n = 3) in triplicate are given as mean percentage ± S.D.

### Lipid peroxidation (LP) evaluation

After treatment with PUVA, cells were trypsinized and collected. Suspensions with approximately 1,5×10^6^ cells ml^−1^ were centrifuged (8000 rpm for 5 min) and the pellet was suspended in 0,5 ml of PBS and extracted twice in chloroform/methanol (2∶1, v:v). Measurement of LP was assessed according to the thiobarbituric acid (TBA) method [38] with slight modifications. The spectrum was recorded in the 400–600 nm range showing a maximum at 532 typical for the MDA-TBA complex. Optical density at 532 nm was corrected for background absorption by interpolation. The standard curve was constructed using 1,1,1,3-tetraethoxypropane, after hydrolysis with 1% H_2_SO_4_, as external standard. The levels of lipid peroxides were expressed as nmol of TBA reactive species (TBARS)/mg protein. The results are the means of three different assays performed in each donor (n = 3).

### Analyses of cell membrane cholesterol and oxysterols

HDFs were suspended extracted with methanol containing BHT 100 µM and 5-α-cholestane 100 ng (Sigma-Aldrich, Milan, Italy) as internal standard. Cholesterol (CH) was measured by GC-MS as previously described [39]. Selected ion monitoring (SIM) was carried out by monitoring m/z 329 and 458 for CH, 454 for 7β-OH-cholesterol (7β-OH-CH), 456 for 7-keto-cholesterol (7-keto-CH), 217 and 357 for 5α-cholestane (IS). The mean value of experiments performed in each donor (n = 3) in duplicate is expressed as microgram (for CH) or as nanogram (for 7β-OH-CH and 7-keto-CH) for per milligram of proteins ± S.D.

### Western Blot analysis of cell cycle proteins

Samples were lysed in RIPA buffer with protease inhibitors. Aliquotes of cell proteins (30 µg) were resolved on SDS-polyacrilamide gel and transferred to nitrocellulose membrane and then treated with anti-p53 (clone DO-1, Dako, Milan, Italy; diluted 1∶3000 in TBS-T), anti p21 (Santa Cruz Biotechnology Inc., Santa Cruz, CA, USA; diluted 1∶3000 in TBS-T), anti phospho-p38 (Cell Signaling Technology Inc., Danvers, MA, USA; diluted 1∶3000 in TBS-T), or anti IκB-alpha (Santa Cruz Biotechnology Inc., Santa Cruz, CA, USA; diluted 1∶1000 in TBS-T) overnight at 4°C. Horseradish-peroxidase-conjugated goat anti-mouse or anti-rabbit immuglobulins (Santa Cruz, Biotechnology Inc., Santa Cruz, CA, USA) were used as secondary antibodies. Antibodies complexes were visualized using the ECL Chemiluminescence Luminol Reagent (Santa Cruz Biotechnology Inc., Santa Cruz, CA, USA). As a loading control, the blots were reprobed with an anti-β-tubulin or anti- glyceraldehyde-3-phosphate dehydrogenase (GAPDH) antibody (Sigma-Aldrich, Milan, Italy).

### RARE Transfection and luciferase assays

Cells were plated in a 24-well plate at a density of 2×10^4^ cells/well and left to grow overnight. Afterwards cells were transfected with retinoid responsive element (RARE) reporter, negative control and positive control (CignalTM RARE Reporter Assay Kit; Superarray Bioscience Corp., Frederick, USA). After 24 h, cells were treated with 5µM ReOH for 6 h, 5µM AtRA for 6–48 h, and 4µM Octa for 6–48 h. Measurement of luciferase activity was carried out at the end of the treatments. Cells were harvested in 100 µl of lysis buffer and soluble extracts assayed for luciferase and Renilla activities by using Dual-Luciferase Reporter Assay System (Promega Corp., Madison, USA) according to the manufacturer's procedure.

### RNA extraction and real time RT-PCR

Total RNA was isolated using an RNeasy Mini kit (Qiagen, Hilden, Germany). Following DNAse I treatment, cDNA was synthesized from 1 µg of total RNA using ImProm-II Reverse Transcriptase (Promega Corporation, Madison, WI) according to the manufacturer's instructions. Real time RT-PCR was performed with SYBR Green PCR Master Mix (Bio-Rad, Hercules, CA) and 200 nM concentration of each primer. Sequences of all primers used are indicated in Table S1. Reactions were carried out in triplicates using the Real-Time Detection System (iQ5 Bio-Rad, Milan, Italy) supplied with iCycler IQ5 optical system software version 2.0 (BioRad). The thermal cycling conditions comprised an initial denaturation step at 95°C for 3 minutes, followed by 40 cycles at 95°C for 10 seconds and 60°C for 30 seconds. Levels of gene expression in each sample were quantified applying the 2^−ΔΔC^_T_ method, using GADPH as an endogenous control.

### PPARγ transactivation assay

HDFs were transfected with pGL3-(Jwt)3TKLuc reporter construct [40] using Amaxa human fibroblasts Nucleofector kit (Lonza, Basel, Switzerland) according to the manufacturer's instructions. Twenty-four and forty-eight hours after treatment with PUVA ± Octa, cells were harvested and assayed for luciferase activity using Promega's Dual Luciferase (Promega) according to the manufacturer's protocol. The renilla luciferase plasmid was also transfected as an internal control for monitoring transfection efficiency and for normalizing the firefly luciferase activity. The mean value of luciferase activity performed in each donor (n = 3) in duplicate is expressed as fold of the activity ± S.D. obtained in cells treated divided by luciferase activity from non-stimulated cells.

### RNA interference experiments

For the RNA interference experiments, HDFs were transfected with 100 pmol (h) siRNA specific for PPARγ (sc-29455; Santa Cruz Biotechnology). An equivalent amount of non-specific siRNA (sc-44234; Santa Cruz Biotechnology) was used as a negative control. Cells were transfected using the Amaxa human fibroblasts Nucleofector kit (Lonza) according to manufacturer's instructions. To ensure identical siRNA efficiency among the plates, cells were transfected together in a single cuvette and plated immediately after nucleofection. Twenty-four hours following transfection, HDFs were treated with PUVA and post-incubated with 2µM Octa in agreement with the experimental design.

### Statistical analysis

Statistically significant differences were calculated using Student's *t*-test. The minimal level of significance was p≤0.05.

## Results

### Identification of a specific PPARγ modulator as a useful tool to study possible interference with PUVA-induced damage

**Figure 1:**
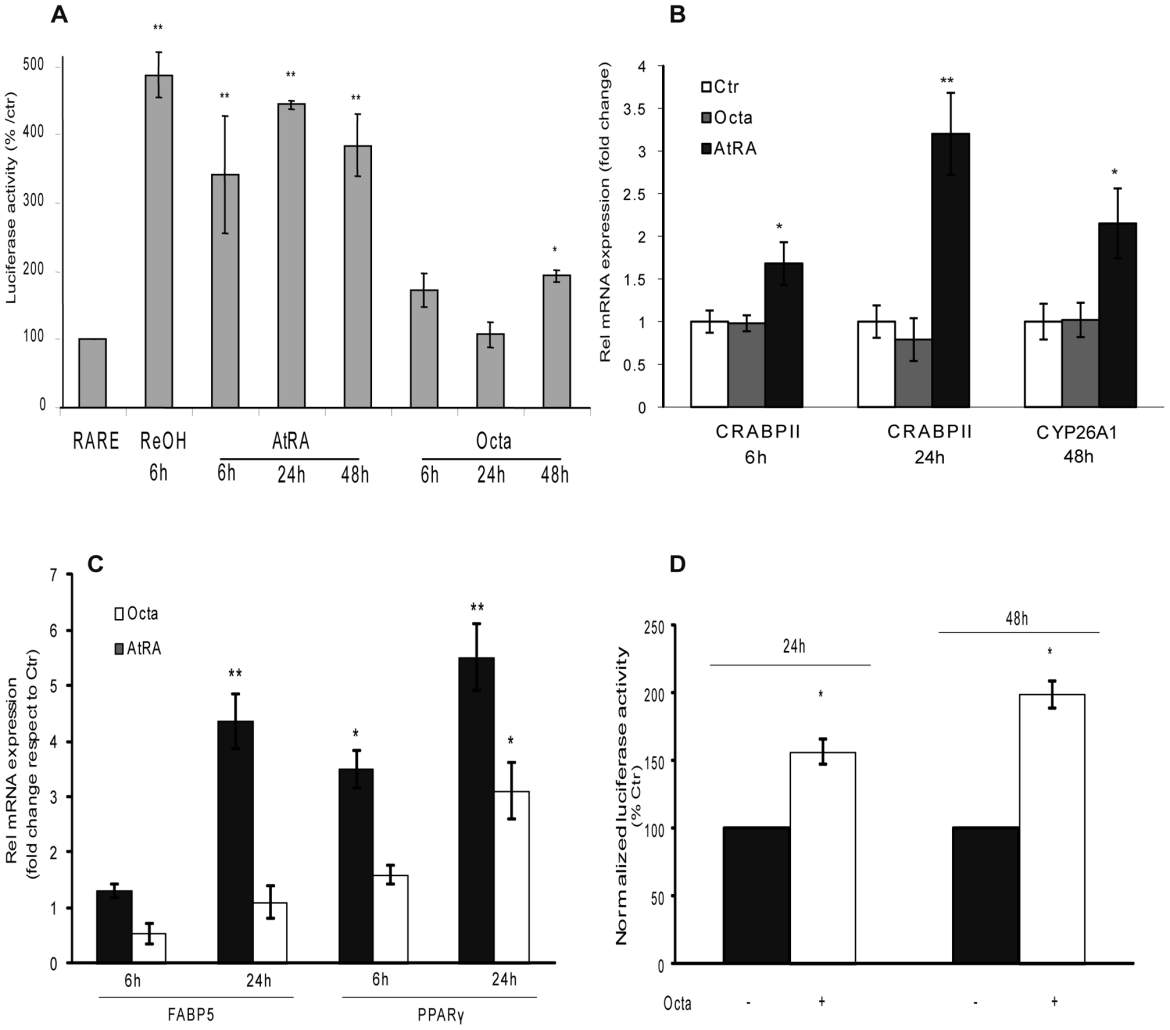
Evidence for Octa-mediated activation of PPARγ-linked signal transduction. (A) Activation of RARE. Cells (2×10^4^cells/well) were plated in a 24-well plate and after 24 h they were transfected with RARE. After 24 h, cells were treated with 5µM ReOH for 6 h, 5µM AtRA for 6–48 h, and 2µM Octa for 6–48 h. Measurement of luciferase activity was assessed as reported in **Materials and Methods**. (B) Quantitative real-time RT-PCR was performed to measure the expression of CRABPII and CYP26A1 mRNA at various time points after treatment with 2µM Octa or 5 µM AtRA. The values were normalized to GAPDH mRNA levels. (C) Quantitative real-time RT-PCR was performed to measure the expression of FABP5 and PPARγ mRNA at various time points after treatment with 2µM Octa or 5µM AtRA. The values were normalized to GAPDH mRNA levels. (D) Luciferase activity analysis of cells transfected with pGL3-(Jwt)3TKLuc reporter construct. After 24 h of transfection, cells were treated with 2µM Octa. The measurement of luciferase activity was carried out 24 h and 48 h after treatment. *p<0.05; **p<0.001 respect to untreated control cells.

To investigate the role of PPARγ modulation in PUVA-SIPS, we used Octa, a compound we previously reported to activate PPARγ in human melanocytes [31]. Because the chemical structure of Octa resembles the polyene chain of carotenoids, we evaluated the ability of this molecule to modulate the retinoid-mediated signaling in HDFs to study the activation of retinoic acid receptor (RAR) and the subsequent transcriptional activation of RARE. We compared the effects with those caused by the specific retinoid receptor ligands AtRA and ReOH. Both ReOH and AtRA induced an early (6 h) and relevant enhancement of the expression of the RARE-driven reporter, whereas Octa showed a mild capacity to transactivate RARE only after 48 h (Fig. 1A). Moreover, Octa treatment did not exhibit any ability to induce the mRNA expression of cellular retinoic acid binding protein 2 (CRABPII) or cytochrome P450 hydroxylase (CYP26), two genes that contain RARE reporter promoters, which are directly involved in the proliferative response elicited by retinoid-like molecules, whereas atRA induced a relevant up-regulation of both genes (Fig. 1B). In contrast, Octa was more effective than atRA in inducing the expression of PPARγ and fatty acid binding protein-5 (FABP5), a carrier protein for PPAR ligands, at the evaluated time points (6 and 24 h) (Fig. 1C). Consistently, a luciferase assay using the pGL3-(Jwt)TKLuc reporter construct [40] showed that Octa enhanced luciferase expression at 24 and 48 h (Fig. 1D).

### PUVA induced a significant reduction of PPARγ expression and activity

**Figure 2:**
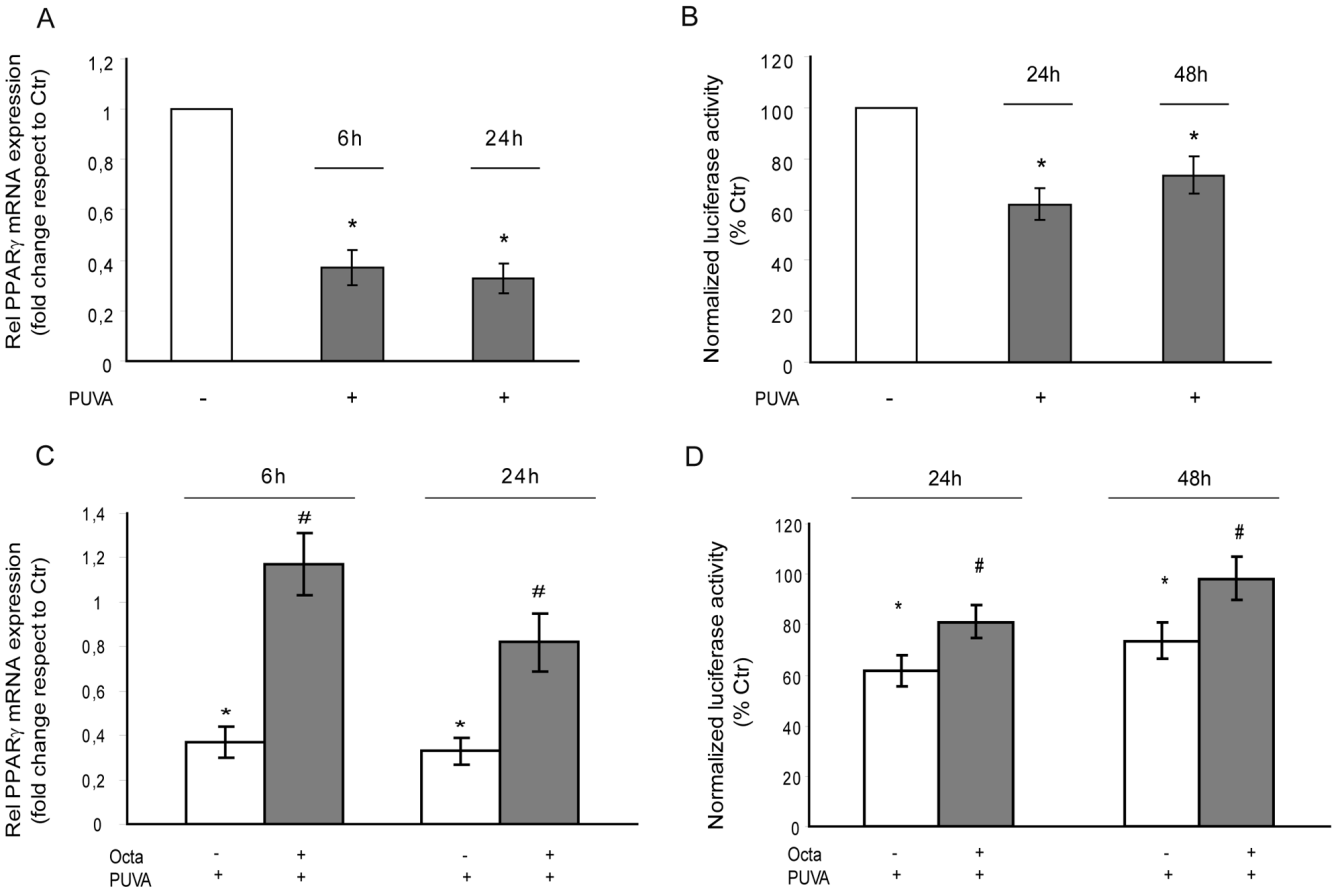
Evaluation of PUVA induced effects on PPARγ expression and activity. (A) Real-time RT-PCR was performed to measure the expression of PPAR-γ mRNA 6 h and 24 h after PUVA exposure. The level of PPAR-γ mRNA was normalized to the expression of GAPDH and is expressed relative to untreated control cells (*p<0.05 respect to Ctr). (B) Luciferase activity analysis of cells transfected with pGL3-(Jwt)3TKLuc reporter construct. After 24 h of transfection, cells were treated with PUVA. The measurement of luciferase activity was carried out 24 h and 48 h after treatment (*p<0.05 respect to Ctr). (C) Real-time RT-PCR was performed to measure the effect of Octa post-treatment on the expression of PPAR-γ mRNA 6 h and 24 h after PUVA exposure. The level of PPAR-γ mRNA was normalized to the expression of GAPDH and is expressed relative to untreated control cells (*p<0.05 respect to Ctr; ^#^p<0.05 respect to PUVA). (D) Luciferase activity analysis of cells transfected with pGL3-(Jwt) 3TKLuc reporter construct. After 24 h of transfection, cells were treated with PUVA and post-incubated with Octa. The measurement of luciferase activity was carried out 24 h and 48 h after treatment (*p<0.05 respect to Ctr; ^#^p<0.05 respect to PUVA).

A reduction of PPARγ expression in H_2_O_2_-SIPS HDFs has been reported to reflect age-related inflammation and aging progression [41]. We previously demonstrated that azelaic acid, a natural compound that is able to act as a ligand of PPARγ, was able to revert, at least in part, PUVA-induced decrease in PPARγ activation [42]. To confirm that PPARγ represents a main biological target of PUVA-SIPS, we performed photo-irradiated HDFs RT-PCR analysis and luciferase assay to evaluate the changes in PPARγ expression and/or activity induced by PUVA treatment. Our results showed that PUVA exposure induced an early reduction of PPARγ expression (at 6 and 24 h) as well as a significant decrease in transcriptional activity (at 24 and 48 h) (Fig. 2A and 2B). Octa treatment significantly counteracted the decreased expression and inhibition of PPARγ (Fig. 2C and 2D).

### PUVA-induced ROS production and mitochondria damage are counteracted by PPARγ modulation

**Figure 3:**
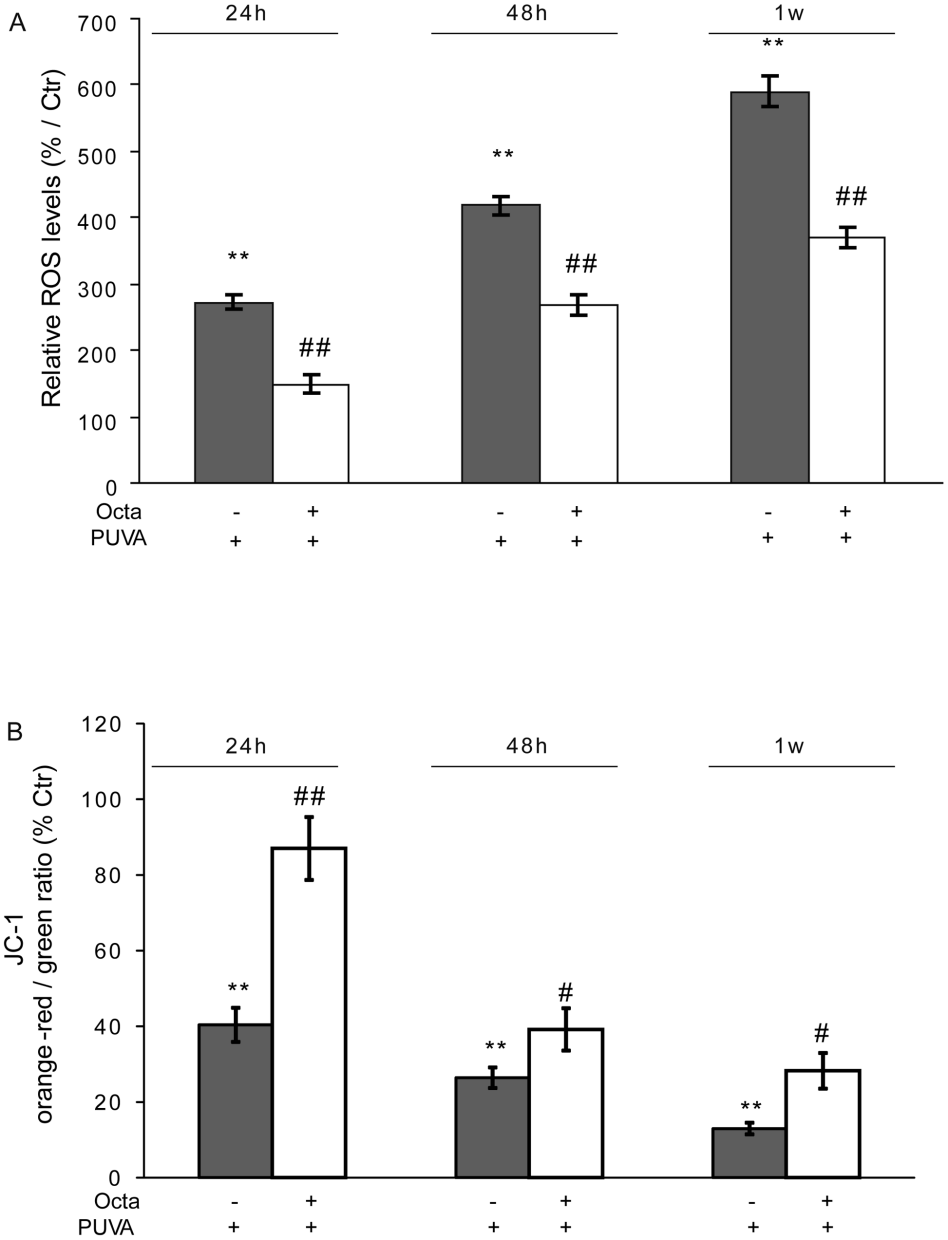
Effects of PPARγ modulation against PUVA-induced intracellular ROS accumulation and mitochondria damage. HDFs were treated with PUVA or left untreated (Ctr). Immediately after irradiation PBS was replaced by fresh medium with or without Octa 2µM for 24 h, 48 h or 1 week. (A) Intracellular oxidative stress was assessed by Flow cytometry using the fluorescent probe DCFH_2_-DA. The median value of fluorescence was used to evaluate the intracellular content of DCF as a measure of the ROS formation. (B) ΔΨ_m_ was assessed in live HDFs using the lipophilic cationic probe JC-1. For quantitative fluorescence measurements, cells were rinsed once after JC-1 staining and scanned with a Flow cytometer **p<0.001 statistically different from unirradiated cells; ^##^p<0.001 compared with PUVA-treated fibroblasts.

Exposure of HDFs to PUVA induces mitochondrial membrane damage with a persistent intracellular ROS accumulation [17]. To determine whether PPARγ modulation has a protective effect, ROS generation was determined at 24 h, 48 h, and 1 week after PUVA exposure using the DCFH_2_-DA assay. PUVA led to a significant time-dependent ROS increase in HDFs, and post-incubation with Octa significantly decreased (p<0.01) ROS production at all evaluated time points (Fig. 3A). ROS generation was correlated with a decrease in mitochondrial ΔΨ_m_ based on JC-1 staining. Consistent with the literature [43], PUVA determined a progressive decline in the ratio of orange-red/green fluorescent JC-1 density compared with sham-irradiated fibroblasts after 24 h, 48 h, and 1 week. PPARγ modulation induced a significant improvement of mitochondrial ΔΨ_m_ (Fig. 3B).

### PPARγ modulation counteracted the imbalance of the redox system in PUVA-treated HDF

**Figure 4:**
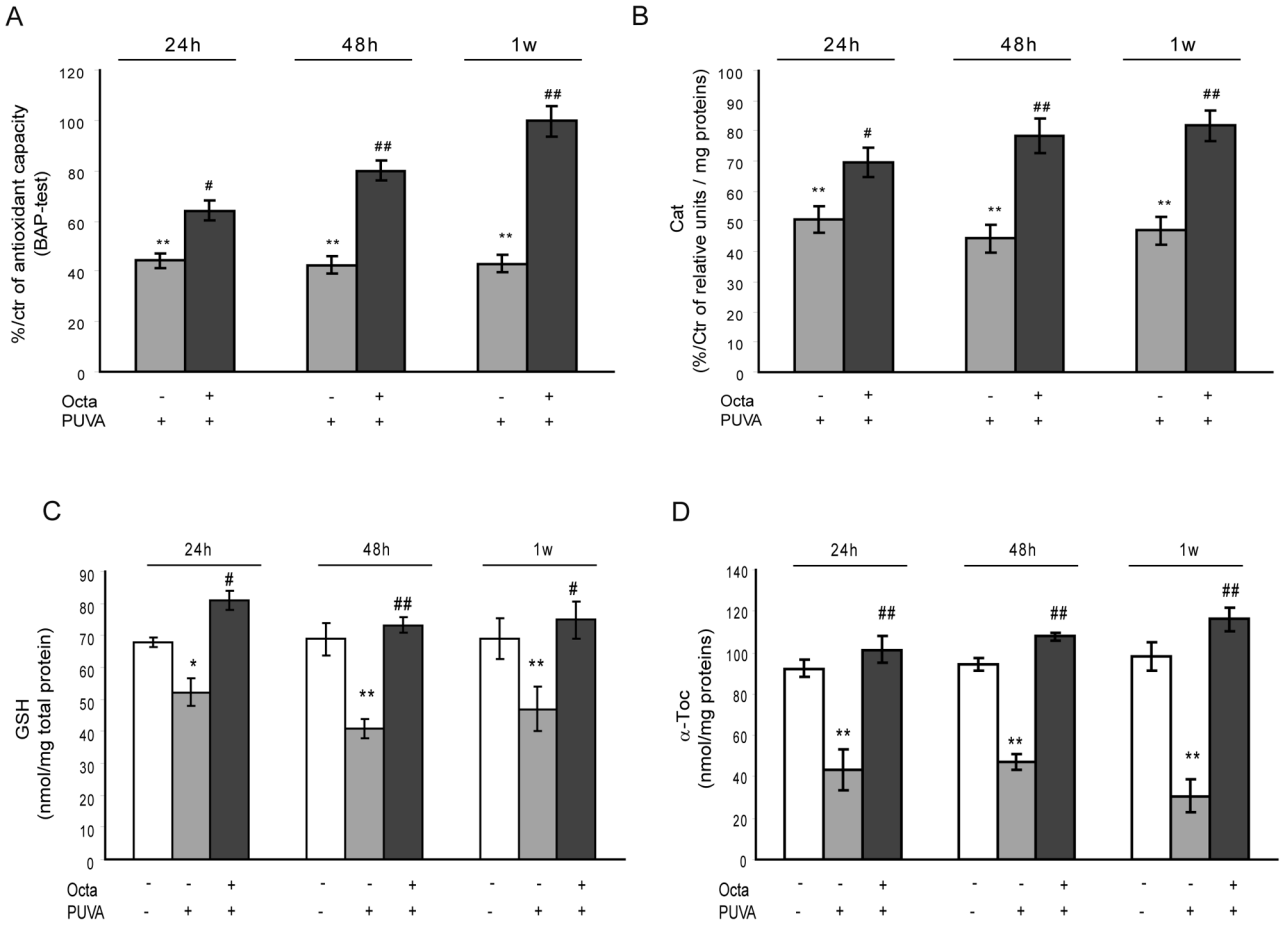
Protective action of PPARγ modulation on PUVA-induced imbalance of cell antioxidant system. HDFs (1×10^6^) were lysed in PBS and protease inhibitor cocktail. Cell lysates were used for analytical determinations. (A) Total antioxidant capacity (TAC) was assessed by BAP-test as described under **Materials and Methods** section. (B) Cat enzyme activity was determined by spectrophotometry as described under **Materials and Methods**. (C) GSH concentrations were determined by HPLC-MS as described in **Materials and Methods**. (D) α-Toc is measured by GC-MS as described in **Materials and Methods**. *p<0.05; **p<0.001 respect to control fibroblasts; ^#^p<0.05; ^##^p<0.001 compared with PUVA-treated fibroblasts.

The PUVA-induced imbalance in the intracellular redox environment was investigated by analyzing the following: a) BAP, an index of overall antioxidant status; b) Cat activity, which is directly involved in the persistent accumulation of hydrogen peroxide in senescent cells; c) GSH, a major endogenous antioxidant; and d) α-Toc, which protects the cell membrane lipid layer by acting as a chain anti-breaking antioxidant. Because our aim was to investigate the ability of PPARγ modulation to interfere with already activated cell senescence, we did not incubate fibroblasts with Octa before PUVA exposure and we considered untreated fibroblasts as the controls. Up to 1 week, PUVA caused a decline in BAP (p<0.01), GSH levels, and α-Toc content, which were recovered by post-treatment with Octa (Fig. 4A, 4C, 4D). Moreover, PUVA led to a relevant and long-lasting decrease in Cat activity to 50% of the baseline value after 24 h, which was still reduced to 47% after 1 week. Octa protected against enzyme damage, leading to a recovery of Cat activity within 1 week (Fig. 4B).

### PPARγ activation is needed to promote cell antioxidant defense

**Figure 5:**
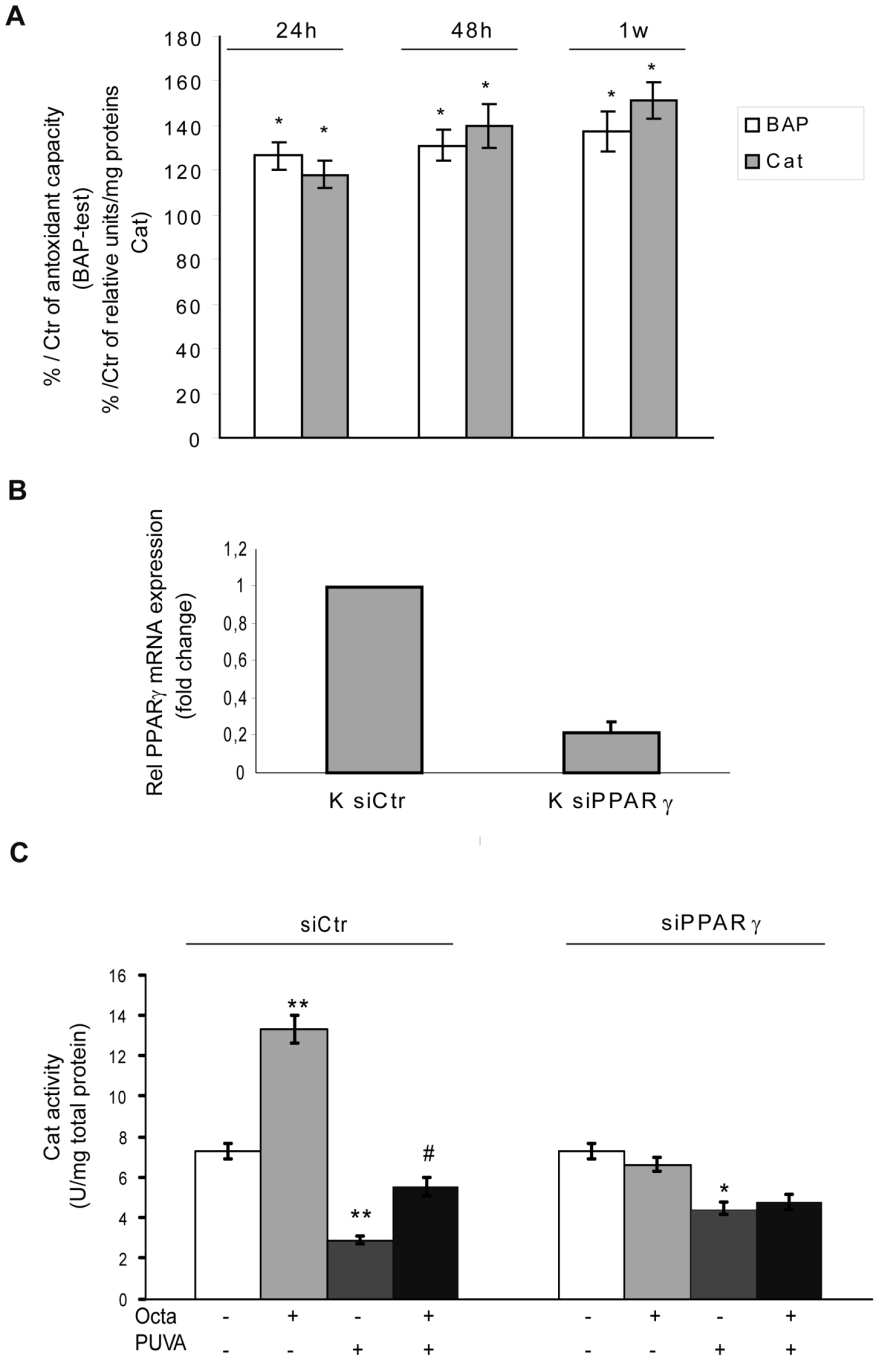
Evidence for PPARγ-induced promotion of cell antioxidant defence. (A) Octa treatment for 24 h, 48 h and 1 week determined a significant increase of antioxidant cell response. TAC was assessed by BAP-test and Cat enzyme activity was determined by spectrophotometry as described under **Materials and Methods** section. (B) HDFs were transfected with siRNA specific for PPARγ (siPPARγ) or non-specific siRNA (siCtr). PPARγ level was evaluated by real-time RT-PCR (C) The activity of Cat was assessed in HDFs transfected with siPPARγ or siCtr and exposed to 2µM Octa for 6 h. In parallel Cat activity was measured in HDFs transfected with siPPARγ or siCtr and exposed to PUVA w/o post-incubation with 2µM Octa. *p<0.05; **p<0.001 respect to control fibroblasts; ^#^p<0.05 compared with PUVA-treated fibroblasts.

In parallel, we treated non-irradiated fibroblasts with Octa to evaluate its capacity to enhance basal antioxidant defense. Endogenous antioxidants and, in particular, total antioxidant capacity and Cat activity in sham-irradiated cells were significantly increased by supplementation with Octa (Fig. 5A). Considering that PPARγ regulates the expression of catalase via functional PPREs identified in its promoter [44], we investigated the implication of PPARγ in the activation of this endogenous antioxidant by Octa in both sham-irradiated and PUVA exposed HDFs, that were transiently transfected with PPARγ siRNA (siPPARγ) (Fig. 5B). As expected, Octa significantly increased Cat activity in siCtr cells but failed to up-regulate Cat in PPARγ-silenced HDFs (Fig. 5C). Furthermore, in PPARγ-deficient HDFs, Octa failed to counteract the decrease in Cat activity caused by PUVA (Fig. 5C), indicating that the increase in the antioxidant enzyme was PPARγ dependent.

### Possible interference of PPARγ against the PUVA-induced modulation of the cellular stress response system

**Figure 6:**
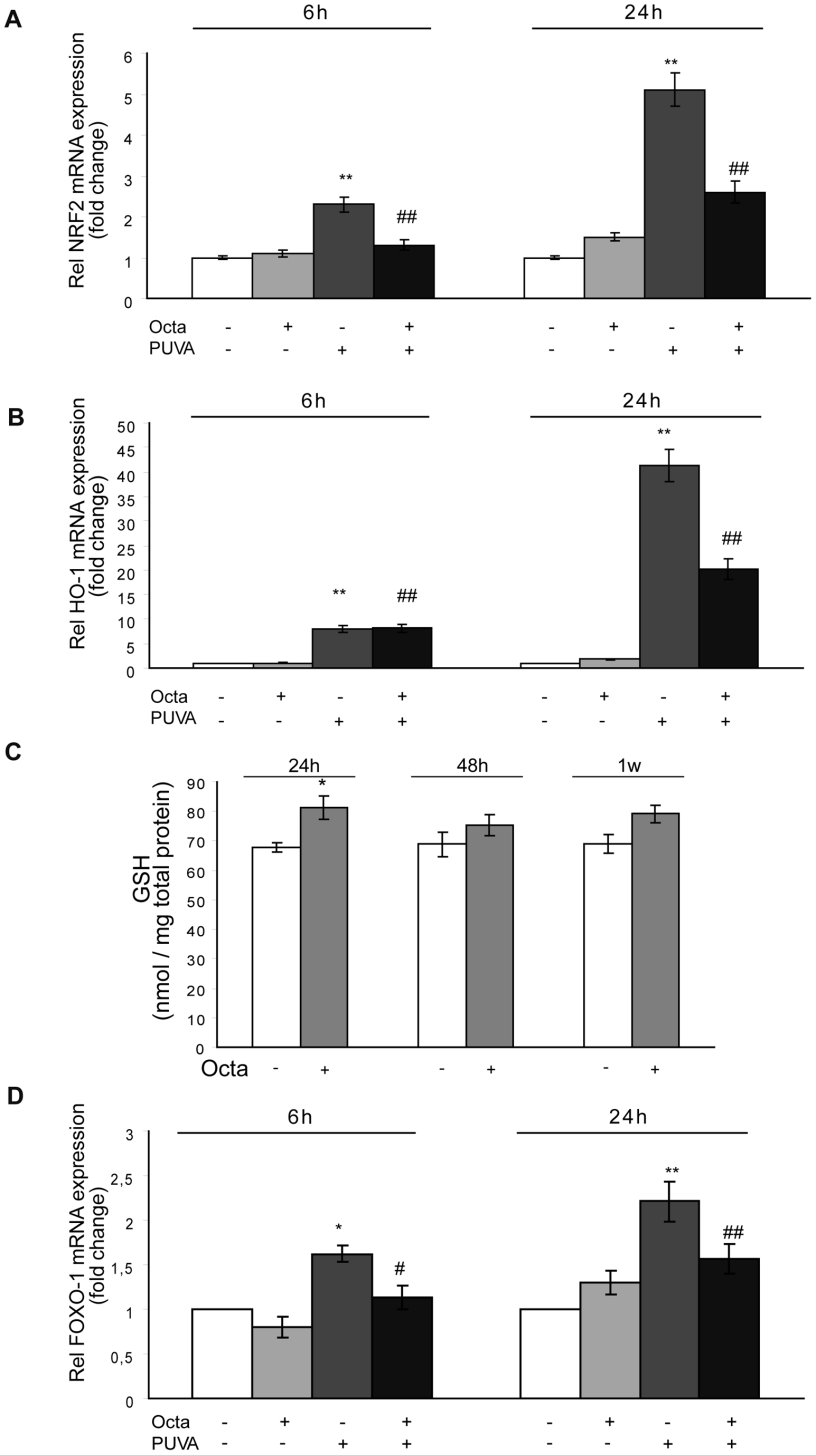
Possible interference of PPARγ against PUVA induced modulation of the cellular stress response system. (A) RT-PCR was performed to measure the expression of NRF2 mRNA 6 and 24 h after PUVA exposure, w/o Octa post-incubation. The level of NRF2 mRNA was normalized to the expression of GAPDH and is expressed relative to untreated control cells (**p<0.001 respect to Ctr; ^##^p<0.001 compared with PUVA-treated fibroblasts). (B) RT-PCR was performed to measure the expression of HO-1 mRNA 6 and 24 h after PUVA exposure, w/o Octa post-incubation. The level of HO-1 mRNA was normalized to the expression of GAPDH and is expressed relative to untreated control cells (**p<0.001 respect to Ctr; ^##^p<0.001 compared with PUVA-treated fibroblasts). (C) GSH concentrations were determined by HPLC-MS) as described in **Materials and Methods** (*p<0.05 respect to control fibroblasts). (D) RT-PCR was performed to measure the expression of FoxO1 mRNA 6 and 24 h after PUVA exposure, w/o Octa post-incubation. The level of FoxO1 mRNA was normalized to the expression of GAPDH and is expressed relative to untreated control cells. *p<0.05 respect to control fibroblasts; ^#^p<0.05 compared with PUVA-treated fibroblasts.

The activation of nuclear factor erythroid-related factor 2 (NRF2) and subsequent induction of NRF2-dependent genes are part of an efficient adaptive response mechanism to electrophilic and oxidant stress, as occurs upon UVA irradiation [45]. Quantitative PCR results indicated that the copy of the cellular NRF2 mRNA increased 2.3 and 5.1-fold, 6 h and 24 h, respectively, after PUVA treatment (Fig. 6A). Cells supplemented with Octa after PUVA exposure showed a significant reduction (p<0.01) of NRF2 mRNA, and no significant modifications of basal level of NRF2 mRNA were observed in HDFs treated with Octa (Fig. 6A). Moreover, NRF2 plays a key role in the UVA-induced up-modulation of heme oxygenase 1 (HO-1), which is considered an immediate cellular response to oxidative insults [46], [47]. However, whereas modest HO-1 expression is cytoprotective, the exacerbation of oxidative injury correlates with high HO-1 expression [48]. In HDFs, the basal level of HO-1 expression was low and PPARγ modulation did not induce relevant changes (Fig. 6B). In response to PUVA, HO-1 expression increased significantly in a time-dependent manner up to 40-fold after 24 h (Fig. 6B), indicating a promotion by the persistent oxidative stress, and Octa treatment significantly reduced (p<0.001) this effect.

Consistent with the incapacity of Octa to increase the basal level of NRF2 mRNA, we observed only a slight increase in basal intracellular GSH, which is synthesized by glutamate cysteine ligase, an NRF2-dependent gene (Fig. 6C). As discussed above, PUVA exposure caused a strong and long-lasting GSH depletion and Octa significantly counteracted this effect (Fig. 4C).

The FoxO1 is a transcription factor that is directly involved in cell responses to ROS [49], and it plays a substantial role in skin photoaging [50]. Moreover, a regulatory feedback loop involving PPARγ and FoxO and characterized by a transrepression mechanism has been described [51]. In this set of experiments, PUVA-treated HDFs showed a significant increase in FoxO1a mRNA expression, and PPARγ stimulation was able to reverse this effect (Fig. 6D), suggesting that this molecule promotes an antioxidant defense response by also interfering with the FoxO-induced repression of PPARγ.

### PPARγ modulation reduced the senescence-like phenotype in PUVA-treated HDFs

**Figure 7:**
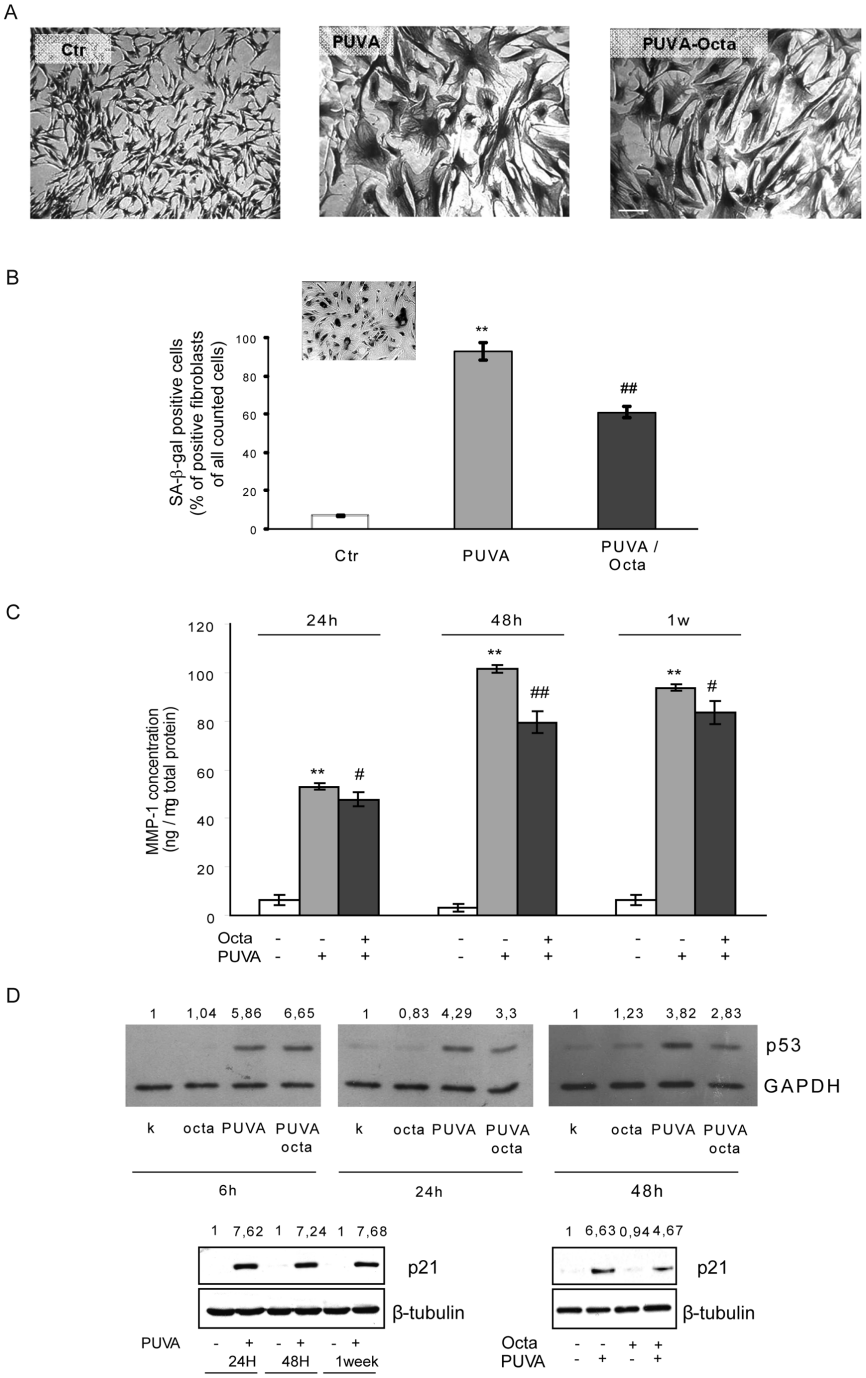
Effect of PPARγ modulation on PUVA-induced expression of senescence-like phenotype in HDFs. After PUVA treatment, HDFs were cultured in the absence or in the presence of 2µM Octa. The medium was changed every 3 days to ensure efficient antioxidant capacity. (A) To evaluate fibroblast morphology, 2 weeks after PUVA in the absence or presence of Octa treatment, cells were fixed and stained with Comassie Brilliant Blue. Scale bar 50 µm. (B) SA-β-gal expression was detected as described in **Materials and Methods**. The *inset* represents fibroblasts after PUVA-treatment revealing a senescent phenotype with enlarged cytoplasmic morphology and SA-β-gal expression. The number of SA-β-gal positive fibroblasts is shown as mean ± SD of three independent experiments. **p<0.001 as compared with mock treated controls; ^##^p<0.001 as compared with PUVA-treated fibroblasts. (C) Supernatants were collected from mock-treated fibroblasts, at 24 h, 48 h and 1 week post PUVA-treatment. MMP-1 release was assessed by ELISA-kit. Three independent experiments in each donor (n = 3) were performed to determine specific MMP-1 protein concentrations in the supernatants. **p<0.001 as compared with mock-treated fibroblasts; ^#^p<0.05; ^##^p<0.001 as compared with PUVA-treated fibroblasts. (D) Total cellular proteins (30µg/lane) were subject to 10% SDS-PAGE. Variation of protein loading was determined by reblotting membrane with an anti-β-tubulin antibody. Western Blot assays are representative of at least three experiments. Increase of p53 and p21 proteins expression is remarkable 24 h after irradiation as well as until 7 days. Octa treatment decreased PUVA-induced expression of p53 protein (at 24 and 48 h) and of its target gene p21 (at 1 week).

We showed that an altered expression and activity of PPARγ is an early effect determined by PUVA and may be implicated in the appearance of the PUVA-induced cell-senescent phenotype. The interference of PPARγ in PUVA-induced cell senescence was also investigated by examining its effect on typical senescence features, such as cell morphology, SA-β-gal expression, MMP-1 release, and regulatory cell cycle protein expression. PPARγ modulation was able to rescue, at least in part, the enlarged and flattened senescent fibroblast morphology observed 4 weeks after PUVA exposure (Fig. 7A). SA-β-gal is a β-galactosidase whose activity is detectable at pH 6.0 in cultured cells undergoing replicative or induced senescence but whose activity is absent from proliferating cells [33]. In HDFs exposed to PUVA, SA-β-gal activity was detected after 1 week followed by a steady increase up to 4 weeks, when virtually all of the fibroblasts exhibited *de novo* activity of SA-β-gal (insert in Fig. 7B). The total number of cells was not significantly different, but the percentage of SA-β-gal-positive fibroblasts was significantly suppressed (approximately 40%) by post-treatment with Octa (Fig. 7B). In HDFs, PUVA induced a strong and persistent release of MMP-1, the main metalloproteinase induced by UV exposure [52], [53], with a maximum at 48 h after photo-irradiation and an approximately 10-fold (SE ± 0.42) higher amount compared to that of non-irradiated control cells (Fig. 7C). Octa caused a mild but significant decrease of MMP-1 release with a maximum reduction of 21% at 48 h (Fig. 7C); however, it had no effect on the basal secretion of MMP-1 (data not shown). Growth arrest is an important feature of cellular senescence and stress-induced premature senescence. We observed a strong expression of p53 and p21 proteins starting from 24 h after PUVA that was still elevated after 1 week (Fig. 7D). p53 and p21 were not detectable in untreated and Octa-treated control cells. Octa significantly reversed the up-regulation of p53 protein expression after 24 h and p21 after 1 week (0.65-fold and 0.7-fold compared to PUVA-treated samples, respectively) (Fig. 7D). In addition, we detected a moderate expression of p16 in PUVA-treated cells, but Octa post-treatment did not induce a significant reduction (data not shown).

### PPARγ modulation interferes with changes in cellular membrane lipids in PUVA-treated HDFs

**Figure 8:**
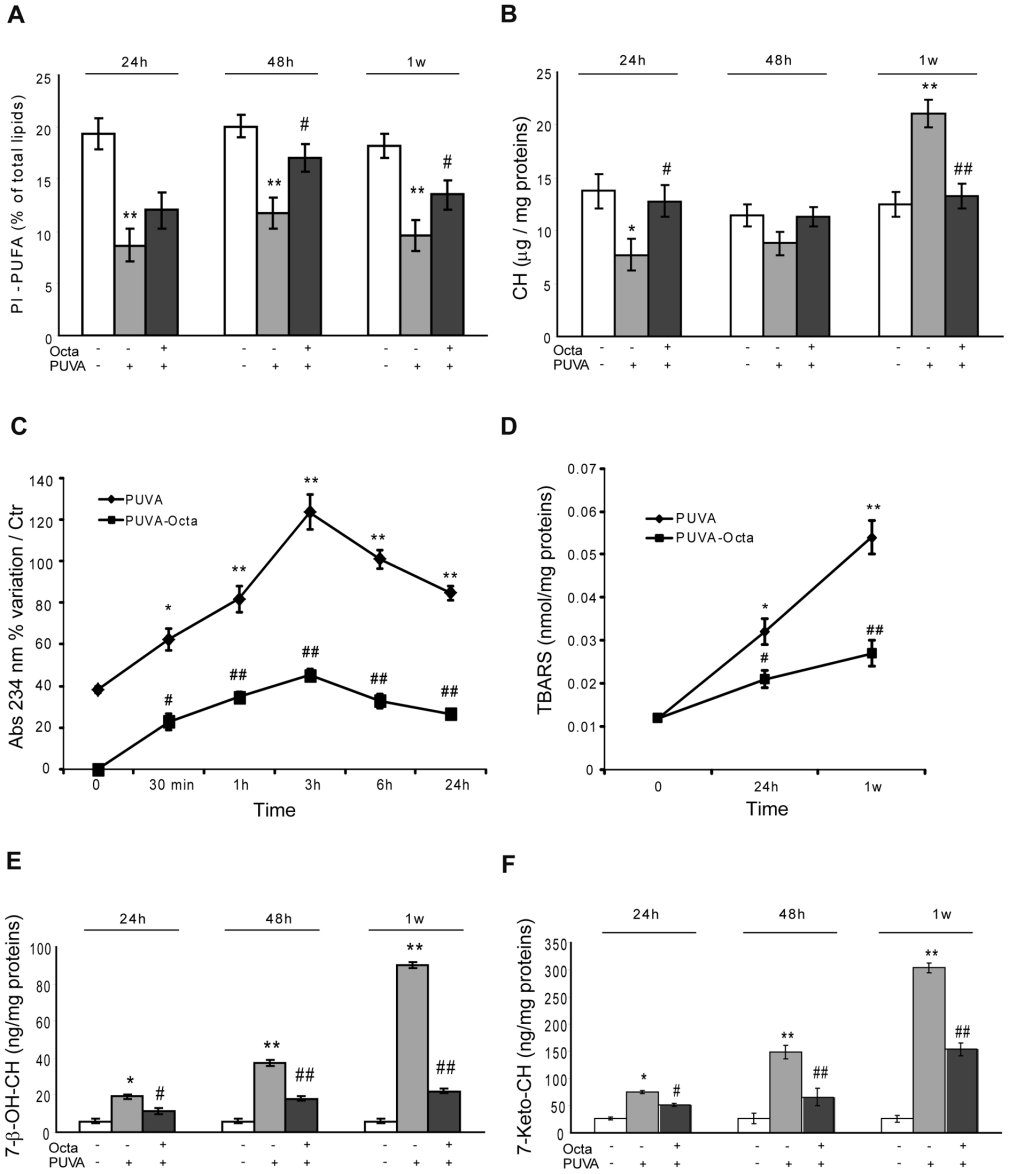
Octa counteracts alteration of lipid cell membrane homeostasis in PUVA treated HDFs. (A) Polyunsaturated fatty acids of membrane phospholipids (Pl-PUFA) in PUVA-treated HDFs were assessed GC-MS as described in **Materials and Methods**. (B) Chol content was analyzed by GC-MS as described in **Materials and Methods**. (C) Early lipid peroxidation products were assessed by the spectrophotometric evaluation of conjugated diene levels as described in **Materials and Methods**. (D) End products of lipid peroxidation were measured according to TBA assay as described in **Materials and Methods**. (E) and (F) Chol oxidation was evaluated by assessing 7β-OH-CH and 7-keto-CH as described in **Materials and Methods**. *p<0.05; **p<0.001 respect to control fibroblasts; ^#^p<0.05; ^##^p<0.001 compared with PUVA-treated fibroblasts.

Unsaturated lipids in cell membranes, including phospholipids and cholesterol, are well-known targets of oxidative modification, which can be induced by a variety of stresses, including UVA-induced photodynamic stress. To evaluate the modifications of the plasma membrane induced by PUVA oxidative damage, we assessed the content of polyunsaturated fatty acids of membrane phospholipids (Pl-PUFA) and the level of CH as the main lipid component of raft domains of cell membranes. PUVA induced a significant modification of the fatty acid composition of cell membrane lipids, with a strong reduction in the Pl-PUFA percentage, which was detectable immediately after irradiation (data not shown) and was still reduced at 1 week (Fig. 8A); this was accompanied a bi-modal alteration in the CH level, with an early (up to 48 h) reduction followed by a relevant accumulation 1 week after photo-irradiation (Fig. 8B). PUVA-induced lipid alterations were almost completely reversed by Octa (Fig. 8A and 8B). Moreover, we evaluated the formation of oxidative products, such as conjugated dienes, fatty acid hydroperoxides, TBARS, and oxysterols (7β-hydroxycholesterol (7-β-OH-CH) and 7-ketocholesterol (7-Keto-CH)), in photo-irradiated cells. PUVA-treated HDFs showed a time-dependent accumulation of lipid peroxidation products. In particular, conjugated dienes were the early products of PUVA-induced lipoperoxidation, and their levels peaked after 3 h (Fig. 8C), whereas both TBARS and oxysterols constantly increased up to 1 week after photo-irradiation (Fig. 8D, 8E, and 8F). Octa significantly reduced the PUVA-induced generation and accumulation of these cell membrane oxidation products (Fig. 8C, 8D, 8E, and 8F). Interestingly, oxysterols were reported to induce the expression of p21 and modulation of the phosphorylation signaling involved in the activation of nuclear factor κB (NF-κB), a transcription factor involved in the induction of pro-inflammatory cytokines. [54]. Considering that these processes are implicated in the aging process and age-related inflammatory responses, we hypothesize that the Octa-mediated reduction of oxysterols plays a key role in the disruption of PUVA-SIPS by Octa.

### PPARγ interfered with the PUVA-induced phosphorylation pathway and NF-kB activation

**Figure 9:**
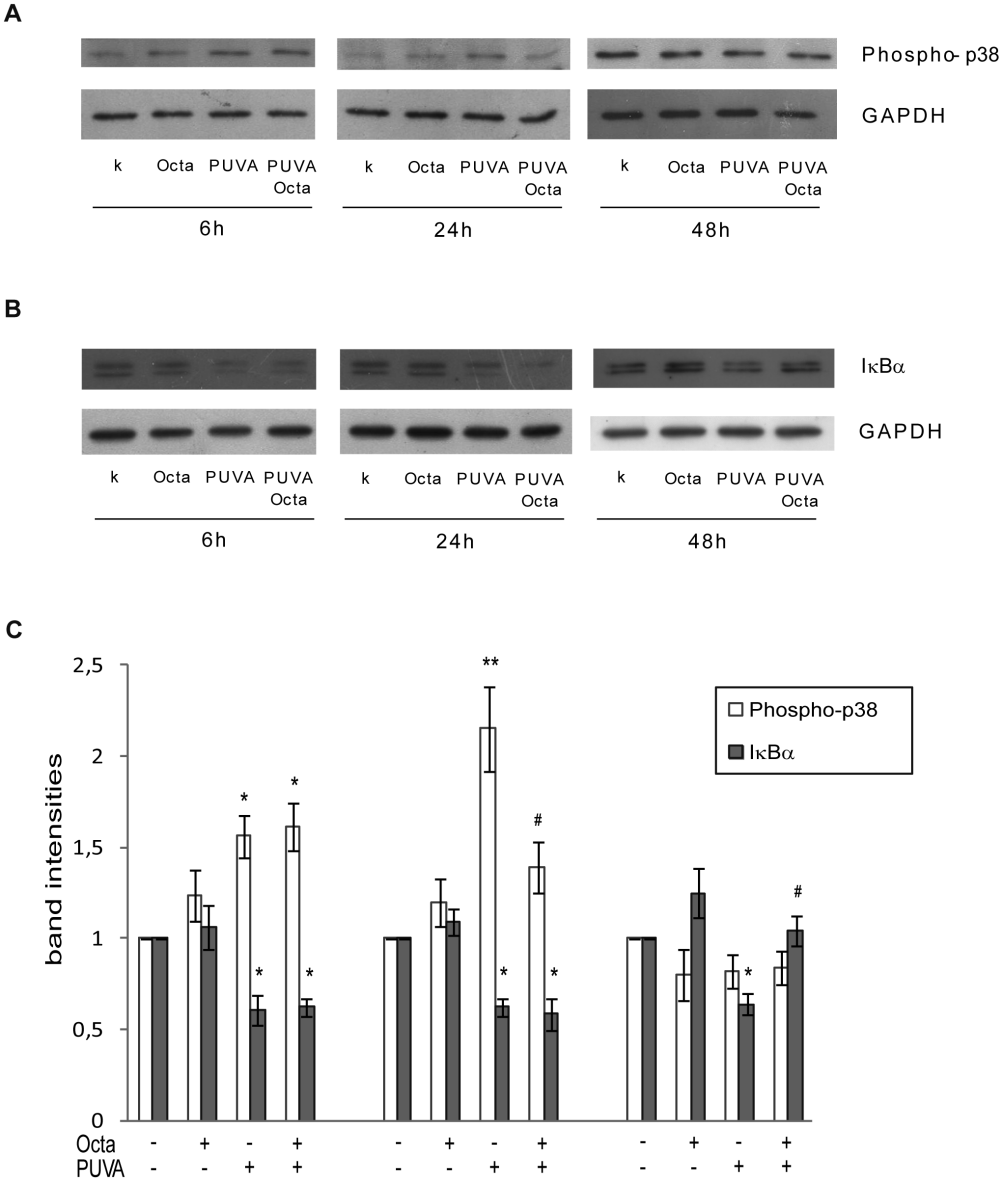
PPARγ interference with PUVA-induced phosphorylation pathway and NF-κB activation. Total cellular proteins (30µg/lane) were subject to 10% SDS-PAGE. Variation of protein loading was determined by reblotting membrane with an anti-GADPH antibody. PUVA-treated HDFs showed an increased phosphorylation of p38 (A) and a decreased expression of IkBα (B). Octa post-treament inhibited p38 phosphorylation (A) as well as decrease of IkBα expression (B) 24 h and 48 h after PUVA treatment, respectively. (C) Densitometric scanning of band intensities obtained from two separate experiments performed in each donor were used to quantify change of protein expression (control value taken as 1-fold in each case). *p<0.05; **p<0.001 respect to control fibroblasts; ^#^p<0.05 compared with PUVA-treated fibroblasts.

The UV-induced inflammatory process in the skin is characterized by ROS-mediated phosphorylation of mitogen-activated proteins kinases (MAPKs), including p38 kinase, and the subsequent activation of NF-κB, To determine the alterations of the phosphorylation pathway and NF-κB activation in the PUVA-SIPS model and the possible protective effect of PPARγ modulation, phosphorylation of p38 and expression of IκBα were evaluated by Western Blot. PUVA-treated HDFs showed an increased phosphorylation of p38 (Fig. 9A) and a decreased expression of IκBα (Fig. 9B), indicating that the activation of the pro-inflammatory response is involved in the senescence-like phenotype. Octa post-treatment inhibited p38 phosphorylation and decreased IκBα expression at 24 h and 48 h after PUVA treatment, respectively (Fig. 9A and 9B). The ability of Octa to interfere with the PUVA-induced activation of the phosphorylation pathway and the activation of NF-κB at later time points compared to its effects on PPARγ activation and generation of cell membrane lipid peroxidation products suggests that p38 and NF-κB are not direct targets of Octa, but they can be modified by the Octa-induced activation of PPARγ and a reduction of the ROS-induced lipoperoxidation process.

## Discussion

PUVA-SIPS is characterized by the persistent induction of ROS and stable alteration of the cell redox system inducing robust aging markers, including morphological changes, increased staining of SA-β galactosidase, and MMP-1 release, thereby representing a suitable tool for the analysis of photoaging-related mechanisms *in vitro* [17]. The imbalance of the antioxidant network is crucial for propagating PUVA-induced oxidative stress, as demonstrated by the ability of antioxidant molecules to counteract the phenomenon [17], most likely not exclusively due to scavenging ROS but also to the modulation of cell signaling pathways.

To further investigate the mechanism mediating the imbalance of the cell-redox system, we focused on possible cell targets and transcription factors involved in the induction of PUVA-SIPS. We previously reported that azelaic acid, a modulator of PPARγ, interfered with PUVA-induced cell responses, and here we sought to determine whether this nuclear receptor represents a “conductor” of PUVA-SIPS. PPARs regulate the expression of genes involved in multiple biological pathways, including cellular lipid metabolism, inflammation, differentiation, and proliferation [18]–[20]. Therefore, these nuclear receptors are possible regulators of mitochondrial functions, inflammatory responses, and antioxidant imbalances observed in premature cell senescence. Reduced activity of the proteins PPARγ coactivator-1α (PGC-1α) and PPARγ coactivator-1β (PGC-1β), which are master regulators of PPARγ, is associated with mitochondrial dysfunction and reduced expression of numerous ROS-detoxifying enzymes [22]. We investigated the possible interplay among PPARγ modulation and the PUVA-induced senescence-like phenotype by employing Octa, a polyunsaturated acid with retinoid-like molecular features. Despite its reported features in common with retinoids [29], the molecule caused only a weak activation of RARE and was not associated with the modulation of RA target genes, such as CYP26, which is a cytochrome P450 isoenzyme that specifically metabolizes RA [55], or CRABPII, which transports retinoids to the nucleus [56]. In contrast, Octa significantly activated PPARγ and FABP5, which is an intracellular protein that binds lipid molecules and transports them to PPARs [57]. Consistent with the results reported for H_2_O_2_-SIPS [41], PUVA-treated HDFs showed an immediate decrease in the expression and activity of PPARγ, indicating a relevant role of this receptor in the biological modifications induced by senescence-like phenotype. Octa mitigated the PUVA effects, indicating that PPARγ modulation may be responsible for the protective mechanism. Because PPARγ promotes mitochondrial function and endogenous antioxidants, we evaluated the effects of the nuclear receptor modulation against PUVA-induced damage to these cellular targets. Mitochondrial oxidative stress, characterized by the reduction of the oxidative phosphorylation efficiency and ΔΨ_m_, promotes the senescence of skin cells both *in vitro* [58] and *in vivo* [59]. In PUVA-treated HDFs, we observed a progressive accumulation of intracellular ROS and a decline in ΔΨ_m_, indicating that mitochondria are involved in the senescence-like phenotype. However, the excessive ROS generation induced by PUVA overwhelmed the cell redox system. Because antioxidant enzymes are themselves targets of oxidative modifications [60], PUVA-SIPS mimics the alterations observed in photoaged cells [61]. In particular, PUVA-treated HDFs showed a dramatic decline in Cat activity and a significant reduction in intracellular GSH, which are both critical for preserving cellular redox balances, with a very low recovery to basal values. Despite the reported antioxidant action of Octa [29], the compound reduced but did not abrogate PUVA-induced intracellular ROS accumulation and the alteration of mitochondrial integrity, suggesting that scavenging ability is only partly involved in the protective effect of Octa. Octa treatment promoted the increase of both Cat activity and GSH levels in both untreated and PUVA-exposed HDFs, interfering with their biosynthetic pathways.

PPARγ is directly involved in the regulation of the expression of Cat via functional PPREs identified in its promoter [44], and the activation of PPARγ by Octa was functionally relevant for the induction of catalase activity, as the use of a specific PPARγ siRNA abolishes this effect. Moreover, silencing the PPARγ receptor significantly reduced the PUVA-induced decrease in Cat activity and completely abrogated the protection of Octa against this damage.

PPARγ regulates antioxidant defense and counteracts mitochondrial damage in close connection with other transcription factors involved in the oxidative stress response [23]. In the activation of cellular defense against the oxidative stress antioxidant response, PPARγ cooperates with NRF2, a transcription factor that regulates the expression of antioxidant genes, including HO-1 and the glutamate cysteine ligase, which is the rate-limiting enzyme for the cellular biosynthesis of GSH [23]. PUVA induced an increased expression of NRF2, indicating the attempt of the cells to activate an adaptive response against oxidative stress. Among the target genes of NRF2, HO-1 acts as a general marker of oxidative stress [47]. The activation by UVA is an emergency stress response that results in the clearance of excess heme levels. However, HO-1 overexpression has deleterious consequences if the excess free heme is not quickly catabolized [48]. The balance of expression is particularly delicate for UVA, which itself damages heme-containing proteins and releases labile iron. Moreover, the induction of HO-1 by the ROS-generating system occurs in association with the depletion of intracellular GSH and may be enhanced by the chemical depletion of GSH [62], [63]. Octa significantly reduced NRF2 and HO-1 mRNA expression in PUVA-treated HDFs, suggesting an attempt to interrupt the persistent activation of detoxifying genes, which may indicate a compromised redox homeostasis in photo-irradiated cells. Although the mRNA expression of NRF2 was increased in photo-irradiated cells, a stable decline in intracellular GSH was observed, whereas Octa effectively counteracted this damage, indicating its ability to promote the maintenance of the NRF2 signaling pathway, leading to the up-modulation of the GSH level. These findings strongly suggest a relationship between NRF2 and PPARγ in the PUVA-induced senescence-like phenotype. However, the mechanisms that regulate the reciprocal feedback circuit between these transcription factors require further investigation. Moreover, PPARγ acts at an intersection of the intracellular signaling pathways activated by FoxO1, a transcription factor that plays a pivotal role in cell fate decisions because it regulates and is regulated by oxidative stress [49]. FoxO1 may modulate PPARγ at the mRNA and protein levels [51], acting as a transcriptional repressor binding to the PPARγ promoter [64] and reducing PPARγ activity through a transrepression mechanism that involves a direct protein-protein interaction [65]. Octa decreases the PUVA-induced nuclear concentration of FoxO1, ROS accumulation, and mitochondrial damage, suggesting an interference with the regulatory feedback loop between PPARγ and FoxO proteins. Moreover, due to their ability to cross talk with the p53 tumor suppressor gene, FoxOs can participate in ROS-induced cell cycle arrest, a typical feature of cell senescence [50]. PUVA activates p53 stabilization, phosphorylation, and nuclear localization as well as the induction of p21 (Waf/Cip1), which is needed for the entry into the growth arrest state [66], [67]. Octa interfered with the increase of p53 and p21, interrupting the positive axis between FoxO1 and cell cycle proteins. The evidence that the molecule did not interfere with immediate (up to 6 h) PUVA-induced ROS generation (data not shown) and p53 expression indicates that scavenger ability is not relevant for Octa interference with the senescence-like phenotype. In contrast, the compound effectively counteracted typical features of PUVA-induced cell senescence, such as enlarged cell shape, the up-modulation of MMPs and the subsequent malfunction of the connective tissue remodeling process, and a steady increase in SA-β-gal expression, suggesting that the up-modulation of PPARγ can effectively contribute to its “anti-senescence” action.

**Figure 10:**
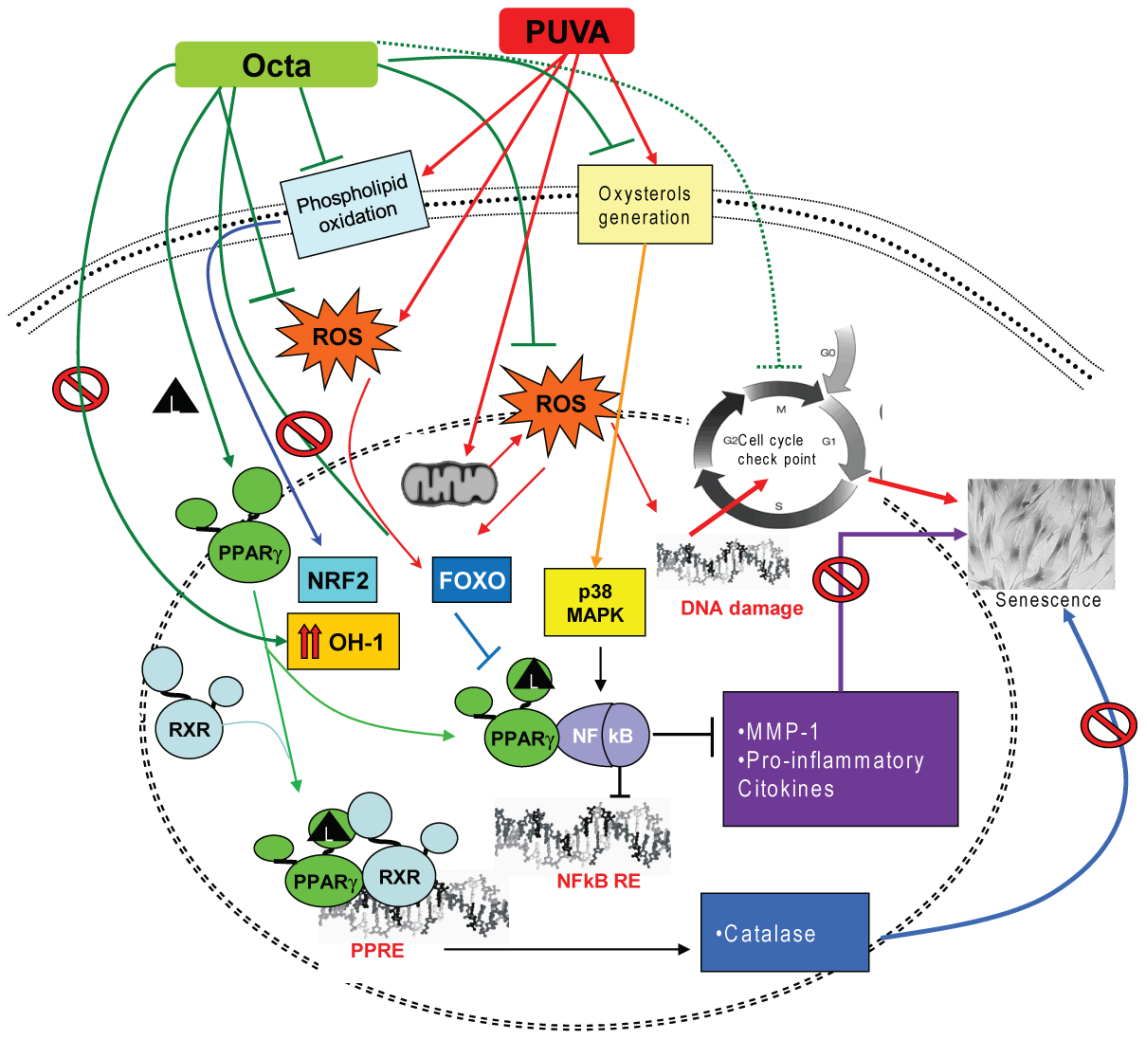
Summary scheme of possible role of PPARγ modulation in counteracting PUVA-SIPS of HDFs. PUVA exposure induced intracellular generation of ROS, alteration of mitochondria function, activation of antioxidant stress response and MAPK phosphorylation pathway, dysregulation of membrane lipid metabolism, DNA-oxidative damage and altered expression of cell cycle regulators. PPARγ modulation by Octa may counteract PUVA-induced senescence-like phenotype. Moreover, Octa ability to reduce phospholipid oxidation and oxysterol generation contributes to the reduction of PUVA-induced inflammatory response and redox imbalance.

Since PPARγ is a key player in lipid metabolism and because damage to cellular lipids is involved in the imbalance of the antioxidant network, we investigated the consequences of PUVA treatment for lipid composition and the possible interference of Octa against this damage. Among the cell compartments, membrane phospholipids play a causal role in the aging process by modulating oxidative stress and molecular integrity [68], [69]. 8-MOP can permeate cell membranes and establish photochemical cross-links between its furan or pyrone ring and unsaturated lipid molecules [70], and the subsequent UVA exposure disturbs the integrity of HDF membrane lipids, as demonstrated by the early and permanent decrease in the Pl-PUFA content and the relevant generation of both early and end-products of lipid peroxidation. The oxidative products of cellular lipids diffuse in the cytosol, interacting with intracellular organelles and determining a propagation of the oxidative stress reaction. Phospholipid oxidation products have been reported to activate NRF2 and HO-1 as a compensatory reaction of cells against oxidative stress [71]. However, the accumulation of lipoperoxidation products induced by PUVA can lead to an excessive over-expression of HO-1, shifting the emergency stress response to a deleterious effect against the cell structure. Therefore, the Octa-induced reduction of PUVA-induced phospholipids oxidation products may contribute to the regulation of NRF2 and HO-1 and the subsequent preservation of cell integrity. In addition to phospholipids, CH plays an indispensable role in regulating the properties of cell membranes and the fluidity and the integrity of lipid rafts [72], [73]. CH accumulation has been observed in fibroblasts obtained from aged skin [74] as well as *in vitro* senescent cells [75]. The pro-oxidant effect of PUVA caused an early decrease in CH and the immediate generation of oxysterols, peroxidation products of CH metabolism representing reliable markers of oxidative stress *in vivo* [76]. Moreover, the stable appearance of the senescence-like phenotype was associated with a time-dependent accumulation of CH and oxysterols. The observed effect of PUVA on CH metabolism prompted us to investigate the role of PPARγ in controlling the activation of the inflammatory response by chronic oxidative stress which is associated with the induction of cell senescence. The age-related inflammatory chronic state has been associated with a reduction of PPARγ function and an increased generation of oxysterols, which act as secondary messengers in MAPK signaling pathways [77], an important component of the pathway that regulates cellular senescence as well as the inflammatory response [78]. PUVA-SIPS was characterized by a progressive generation of oxysterols and the up-modulation of phosphorylation signaling involved in NF-κB activation and, in particular, the increase in phosphorylated p38 and the decrease in IκBα, leading to NF-κB activation. In PUVA-exposed cells, the ability of Octa to counteract the accumulation of oxysterols and the changes in the level of CH may contribute to the observed interference with the phosphorylation pathway. It has been suggested that oxysterols act as signaling molecules [79] by influencing lipid membrane integrity as well as the structure and function of PPAR and RXR receptors and their subsequent modulation of the antioxidant response and inflammation [80]. Therefore, PUVA-SIPS contributes to the identification of how biochemical modulators are integrated in the induction of the chronic inflammation state that is typical of aged skin and provides new insights in the activation of nuclear receptors as novel therapeutic approaches for photo-aging (Fig. 10).

## Conclusions

Taken together, our data suggest that PUVA-SIPS involves a complex interplay of various cellular transcription factors activated by sustained and long-lasting oxidative stress. Mitochondria are the most probable cell targets, and the modulation of PPARγ provides relevant insights into the mechanism of PUVA-SIPS. The reciprocal influences of PUVA-induced signaling pathways have been investigated by employing Octa due to its ability to increase the trans-activation of PPARγ by acting as a partial agonist and interfering with ROS-dependent cellular signaling mechanisms. Interestingly, Octa counteracts certain molecular markers of PUVA-SIPS by improving physiological defense mechanisms without significant changes to the cell redox environment.

## Supporting Information

Table S1 **List of primers used for quantitative real time PCR.** Sequences of primers indicated with an F correspond to sense strands and with an R correspond to anti-sense.
(DOCX)
