# Supplementary material for: Modulation of PPARγ Provides New Insights in a Stress Induced Premature Senescence Model
Source: PLoS One. 2014 Aug 7;9(8):e104045. doi: 10.1371/journal.pone.0104045 (PMC4125176; doi:10.1371/journal.pone.0104045)
Supplement: Table S1 — List of primers used for quantitative real time PCR. Sequences of primers indicated with an F correspond to sense strands and with an R correspond to anti-sense. (DOCX) [file pone.0104045.s001.docx]

**Table S1**

| **Target gene** | **Forward primer** | **Reverse primer** |
| --- | --- | --- |
| hGAPDH | TGCACCACCAACTGCTTAGC | GGCATGGACTGTGGTCATGAG |
| hPPARγ | GCCAAGCTGCTCCAGAAAAT | TGATCACCTGCAGTAGCTGCA |
| hFABP5 | CAGCATCAGGAGTGGGATG | CCTGTCCAAAGTGATGATGG |
| hCYP26A1 | GACATGCAGGCACTAAAGCAAT | CACTGGCCGTGGTTTCGT |
| hCRABP-II | GACCTCGTGGACCAGAGAACTG | CCTGGTGCACACAACGTCAT |
| hNRF-2 | TTCATTGCTACTAATCAGGCTCAG | TGGCTTCTGGACTTGGAACC |
| hHO-1 | CCAGCGGGCCAGCAACAAAGTGC | AAGCCTTCAGTGCCCACGGTAAGG |
| hFOXO-1a | AAGTTCTTGGTGGATGCTCAATC | GCTCGGCTTCGGCTCTTAG |
